# Effects of Ingroup Identification on Ingroup Favouritism during Fairness Norm Enforcement

**DOI:** 10.3390/bs12110415

**Published:** 2022-10-27

**Authors:** Zhen Zhang, Hanli Su, Menghui Li, Hui Zhao, Chunhui Qi

**Affiliations:** Faculty of Education, Henan Normal University, Xinxiang 453007, China

**Keywords:** group identification, ingroup favouritism, fairness norm enforcement, ultimatum game

## Abstract

People tend to voluntarily sacrifice their own interests to reject unfair proposals, and this behaviour is affected by group affiliation. While group bias is a well-established phenomenon, its direction is still unclear, and little attention has been given to possible moderating factors. In two studies, we manipulate participants’ ingroup identification and investigate whether and how individuals with various levels of ingroup identification react differently to unfairness from ingroups and outgroups during an incentivized (Study 1, *N* = 46) and hypothetical (Study 2, *N* = 332) ultimatum game. The results show that participants display a strong preference for their own group. High identifiers tend to accept unfair proposals from ingroups compared to outgroups, whereas this effect is nonsignificant for low identifiers, especially for moderately unfair treatment (offer 7:3). Moreover, higher identification tends to be accompanied by higher ingroup positive expectation, which then leads to greater ingroup favouritism for an offer of 7:3. These results imply that ingroup identification can enhance group favouritism during fairness norm enforcement through ingroup positive expectation.

## 1. Introduction

Social norms are behavioural standards that guide and constrain how people behave in social interactions and maintain their utility by rewarding the good and punishing the bad [1,2]. Respecting and following social norms promote interpersonal cooperation, while ignoring and violating social norms can lead to interpersonal conflict [3]. The resource allocation situation is one of the important areas affected by social norms, and the imbalance of resource allocation is also one of the important reasons for interpersonal conflicts [4,5]. *Fair norm enforcement* refers to the willingness and behaviour of people to voluntarily pay benefits to punish violations of fairness norms [6]. Research based on the ultimatum game has shown that people have a strong preference for fairness, are extremely averse to unfair distribution outcomes and are willing to voluntarily sacrifice their own interests to sanction violators [7,8]. Sanctions for such unfair distribution indicate a social preference for fairness in human society [9], and this tendency might be genetically inherited [10].

Regarding the social context in interpersonal interactions, individuals’ perception and tolerance of injustice are critically affected by interactive partners’ *group affiliation*, which refers to the perception of the identity of the group to which they belong. A large body of studies has investigated the impact of group affiliation on fairness norm enforcement but obtained conflicting results. Some findings found that unfair allocations from ingroups were more likely to be forgiven and accepted by adults and children in both natural groups and artificially induced groups [11,12,13,14,15,16,17,18], which was interpreted as a reflection of ingroup favouritism. Electrophysiological findings found that selfish allocation elicited more positive feedback-related negativity (FRN) than fair allocation between friends [19] and in unintentional group situations [20]. Brain imaging research also implied that people attempted to understand or rationalize wrongdoing from ingroups using mentalizing networks [21,22]. This evidence is in line with social identity theory, which implies that group integration can *promote* individuals’ positive evaluation and favouritism of ingroups, thus making them more inclusive of ingroup perpetrators [5,23,24].

Simultaneously, other research also demonstrated that children and adults were more inclined to sanction selfish ingroup members relative to outgroup members [25,26,27,28,29], which was term as the black sheep effect [30]. Moreover, some electrophysiological evidence has shown that adults exhibit greater differentiated FRN responses to selfish and fair allocation for ingroups than for outgroups, which might reflect a stronger expectation break due to having higher expectations of ingroup players [31,32]. These lines of findings were consistent with norms-focused theory, which suggests that groups serve to nurture cooperative behaviour and that people have a general cooperative expectation and norm towards ingroups [5,15,24]. Violations by ingroups not only *violate* cooperation expectations but also seriously *threaten* group collaboration, ultimately leading to severe sanctions.

Despite these inconsistent results, group affiliations may indeed have an impact on the enforcement of fairness norms, but the direction of this effect remains ambiguous. In two literature reviews, researchers systematically reviewed previous research findings and found that most of them sound consistent with social identity theory [23,24]. In addition, evidence from two meta-analyses also pointed to a small to medium effect size, indicating that children, adolescents and adults preferentially cooperate with ingroup members [33,34]. Moreover, recent studies demonstrated that ingroup bias during fairness norm enforcement was influenced by situational and personality factors [4,20]. Therefore, understanding the intersection and modulator of group affiliation and fairness norm enforcement can provide insight into *when*, *why* and *which* individuals are involved in punishment behaviours.

As a continuation and extension of these pioneering studies, we aim to investigate the role of ingroup identification in moderating the balance between ingroup bias and inequality aversion. *Ingroup identification* refers to the degree to which the ingroup is included in the self [35,36], and could reinforce ingroup favouritism in various social behaviours [37,38,39]. A few recent studies investigating this issue in ultimatum games produced inconsistent results. Kubota et al. (2013) found White Americans accepted more unfair offers from white players than from black players, and this pattern was reinforced by implicit racial bias [12]. In contrast, Mendoza et al. (2014) showed that ingroup violators received more severe punishments than outgroup violators, and this pattern was more pronounced for individuals with greater ingroup identification [26]. Finally, Cram et al. (2018) found ingroup loyalty had nothing to do with ingroup bias [40]. Upon analysing the research findings above, we found that all three studies manipulated and formed group memberships based on natural group identity (i.e., race, college affiliation or territorial identity). Natural group relations are more consistent with real-life interactions and make these studies ecologically valid. Nevertheless, natural group relationships are relatively complex and impure, involving social distance, interpersonal similarity, trust and many other factors, which might result in inconsistencies in these studies. To overcome this shortcoming, the *minimal group paradigm* (MGP) [41] can be used to generate artificial group identity. Furthermore, it is important to note that these research studies only measured the participants’ perception of ingroup identification, and did not manipulate their ingroup identification. The use of correlation methods makes it impossible to determine a causal relationship between ingroup identification and group bias. Finally, the conclusions were less robust and reproducible due to the small sample sizes of the studies. Consequently, more research is necessary to clarify the relationship between ingroup identification and group bias when facing unfair treatment.

To fill this gap, the present study adopts the *minimal group paradigm* (MGP) to temporarily induce participants’ group membership, manipulate their ingroup identification and test the moderation of ingroup identification on the group effect on fairness enforcement. We conduct two studies to test the above issue: one based on laboratory experiments involving an incentivized ultimatum game (*N* = 46) and the other based on observation data from a moderate sample questionnaire involving a hypothetical ultimatum game (*N* = 332). We use college students from our own institution as the participants in both experiments. In light of the overwhelming evidence supporting social identity theory [5,24,33,34], we predict that college students will also have an obvious favouritism for their ingroups when dealing with unfair proposals (H1). Moreover, because Eastern collectivist countries place more emphasis on group interests and prioritize ingroup cohesion [18,42], we also predict that ingroup identification exacerbates ingroup favouritism during allocation scenarios (H2).

## 2. Study 1

In Study 1, we adopt a dot estimation task to induce group affiliation and manipulate participants’ group identification by the levels of prototypicality. After that, we ask participants as responders to perform an incentivized one-shot ultimatum game with ingroup and outgroup players. The purpose of this study is to provide causal evidence to support that identifying with an ingroup can aggravate ingroup favouritism towards ingroup violators.

### 2.1. Materials and Methods

#### 2.1.1. Design and Participants

The study followed a 2 (ingroup identification: high vs. low) × 2 (group affiliation: ingroup vs. outgroup) × 3 (proposal type: 9:1 vs. 7:3 vs. 5:5) mixed factorial design, in which ingroup identification was a between-subjects factor, while group affiliation and proposal type were within-subjects factors. Forty-eight healthy students were recruited for the experiment in exchange for credit and extra task payment. Only female participants were enrolled because males are more parochial during intergroup conflict and cooperation than females [43,44]. The validity of the research would be enhanced if the hypothesis was confirmed among females who are less parochial. The size of the sample was chosen for several reasons. First, an a priori power analysis using G*Power 3.1 was performed to estimate sample size [45]. F-tests and ANOVA (repeated measures and within-between interaction) in G*Power (version 3.1.9.4) were selected. The minimum sample size needed to detect a medium effect (f^2^ = 0.20) was *N* = 44 (22 participants per group) with 0.95 power and 0.05 Type I error rate. Second, a similar three-way interaction showed a medium effect size (partial η^2^ = 0.06) in our recent work [4] when two groups each contain 25 participants. Finally, previous behaviour research on fairness norm enforcement had a similar sample size per condition [46,47]. All participants were right-handed and did not report any psychiatric or neurological disorders. The participants were randomly assigned to either the high or low group identification condition, with 24 participants in each group. Due to the questioning of the validity of the interaction procedure, data for one participant in each group were excluded. Consequently, 23 participants were included in each group. The age differences between the high (*M*_age_ ± *SD*: 20.22 ± 1.04) and low (*M*_age_ ± *SD*: 20.48 ± 0.85) identification groups were not significant: *t*(44) = −0.93, *p* > 0.05. The experimental protocol was approved by the ethics committee of the Faculty of Education, Henan Normal University and adhered to the tenets of the Declaration of Helsinki.

#### 2.1.2. Minimal Group Paradigm and Manipulation of Ingroup Identification

Once arriving at the test room, participants were informed that they had to complete a dot estimation task [48] which aimed to explore the link between spatial perception and analytic ability. According to their performance, they would be allocated to the “specific” or “global” perceivers group. Group affiliation was indicated by a colour cue (blue refers to a specific perceiver group, and red refers to a global perceiver group), and the participants had to wear a badge of the respective colour. The experimenters predetermined the group assignment so that both groups were distributed equally.

Following a previous procedure [49], we used the levels of prototypicality to manipulate the participants’ group identification. In particular, the experimenter orally informed the participants that they were either typical or atypical members of the specific or global perceivers group (high vs. low group identification, respectively).

#### 2.1.3. Manipulation Checks

To check our manipulation of group and group identification, the participants were asked to indicate which group they belonged to (blue or red) and whether they were typical or atypical group members (yes or no). Furthermore, each participant completed the Inclusion of Ingroup in the Self measure (IIS) [36], which consisted of five pairs of circles varying in their degree of interlink, ranging from 1 (*no interlink*) to 5 (*extreme interlink*). The participants were asked to select one out of these pairs that best described their level of identification with their ingroup.

#### 2.1.4. Ultimatum Game

We modified an intergroup version of the ultimatum game that has been used in previous studies [4,17]. In this experiment, all participants played the role of responders. The participants were informed that in each round they would interact with a different anonymous proposer and that these proposers were selected from a pool of previous experiment participants. These proposers were allocated to the red or blue group and made a proposal of 9:1, 7:3 or 5:5. We also notified participants that proposals from previous participants were stored in the computer and a random proposal would be selected by the computer in each round to complete the interaction. In reality, however, the proposers’ allocations were set up by a simple pre-programmed procedure. In a follow-up interview, all participants did not question the experiment’s authenticity. The participants were required to complete two sets of interactions with ingroup members or outgroup members. Throughout each interaction, 15 trials were carried out, with five for each of the three proposals (1, 3, 5 of 10 CNY). During each trial, a colour pie representing the assignment proposal (1500 ms) was presented along with a fixation point (400–800 ms). The participants were required to press the accept or reject button after the blank screen (400–800 ms) by using either their left or right index finger within 1500 ms. When the participant did not indicate a decision within 1500 ms, a new trial was given to enter a valid response. Finally, two coloured images (red or blue) representing both players and their incomes in the current round were displayed on the screen.

In all participants, the sequences of group interactions and keystrokes were counterbalanced. A pseudo-random distribution of trial order was used in each group interaction. Three trials were conducted to practise before the official task. Each participant received a basic payment of 3 Chinese CNY (about USD 0.5) and was told that a monetary reward would be given based on their performance during the task. Finally, the participants received additional rewards ranging from 1.8 CNY to 2.3 CNY (*M* ± *SD*: 2.01 ± 0.17). This study’s dependent variables are the acceptance rates and response time for each proposal in two interactions and the *ingroup favouritism score*, which reflects the difference in the acceptance rates between the intragroup and intergroup contexts [4].

### 2.2. Results

#### 2.2.1. Manipulation Checks

In all cases, participants correctly identified their group membership and whether they belonged to a typical or atypical group. In addition, an independent samples *t* test on the IIS scale scores found that the participants assigned to typical groups reported higher ingroup identification (*M* ± *SE*: 3.57 ± 0.90) than those assigned to the atypical group (*M* ± *SE*: 1.13 ± 1.18), *t*(44) = 7.88, *p* < 0.001. Therefore, we successfully manipulated group affiliation and ingroup identification.

#### 2.2.2. Acceptance Rates

**Figure 1:**
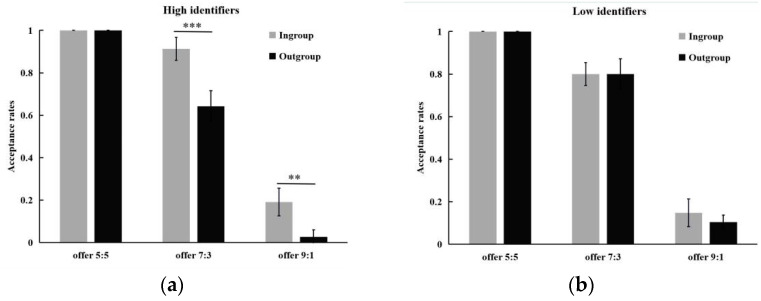
Results from Study 1. Averaged acceptance rates of different proposal as a function of group affiliation for (**a**) high identifiers and (**b**) low identifiers. Error bars indicate standard error. ** *p* < 0.01, *** *p* < 0.001.

A 2 (ingroup identification: high vs. low) × 2 (group affiliation: ingroup vs. outgroup) × 3 (proposal type: 9:1 vs. 7:3 vs. 5:5) ANOVA on the acceptance rate revealed the main significant effects of group affiliation, *F*(1, 44) = 12.85, *p* < 0.001, partial η^2^ = 0.23, and proposal type, *F*(2, 88) = 305.88, *p* < 0.001, partial η^2^ = 0.87. Specifically, higher acceptance rates were linked to intragroup conditions and fairer distribution proposals. The significant interactions of ingroup identification × group affiliation, *F*(1, 44) = 8.60, *p* < 0.01, partial η^2^ = 0.16, and group affiliation × proposal type, *F*(2, 88) = 4.24, *p* < 0.05, partial η^2^ = 0.09, need to be interpreted as part of a significant three-way ingroup identification × group affiliation × proposal type, *F*(2, 88) = 3.86, *p* < 0.05, partial η^2^ = 0.08. A further simple test found that high identifiers were more likely to accept unfair offers: 9:1 (ingroup vs. outgroup: 0.19 ± 0.07 vs. 0.03 ± 0.03) and 7:3 (ingroup vs. outgroup: 0.91 ± 0.05 vs. 0.64 ± 0.07) from ingroup members versus outgroup members, *p*s < 0.01, whereas the acceptance rates of a fair offer 5:5 was unaffected by group affiliation, *p* > 0.05 (see Figure 1a). In contrast, the acceptance rates of all proposals were not influenced by group affiliation for low identifiers, *p*s > 0.05 (see Figure 1b).

#### 2.2.3. Reaction Time

A 2 (ingroup identification: high vs. low) × 2 (group affiliation: ingroup vs. outgroup) × 3 (proposal type: 9:1 vs. 7:3 vs. 5:5) ANOVA on the reaction time found a significant main effect of only proposal type, *F*(2, 88) = 26.09, *p* < 0.001, partial η^2^ = 0.37, indicating that there was a shorter reaction time to an offer of 5:5 (*M* ± *SE*: 637.22 ± 14.03 ms) compared to an offer of 7:3 (*M* ± *SE*: 761.15 ± 20.53 ms) and an offer of 9:1 (*M* ± *SE*: 716.19 ± 16.76 ms), *p*s < 0.05, but the latter two were not significantly different, *p* > 0.05. Neither of the other main effects nor interactions were significant, *F*s < 1.62, *p*s > 0.05.

#### 2.2.4. Ingroup Favouritism Score

Because the participants accepted all fair offers, we calculated the ingroup favouritism score only for the two unfair offers in a linear subtracting way. A 2 (ingroup identification: high vs. low) × 2 (proposal type: 9:1 vs. 7:3) ANOVA on ingroup favouritism score revealed a significant main effect only for ingroup identification, *F*(1, 44) = 8.60, *p* < 0.01, partial η^2^ = 0.16, implying that high identifiers have a higher ingroup favouritism score than low identifiers. Neither the effects of the proposal type, *F*(1, 44) = 0.29, *p* > 0.05, nor the two-way interaction, *F*(1, 44) = 1.69, *p* > 0.05, were significant.

### 2.3. Discussion

Our study successfully induces group affiliation and ingroup identification in female college students and measures their fairness norm enforcement. The results show that female young adults have a strong fairness preference and ingroup favouritism: proposals from ingroup members and those that promote fairness were more likely to be accepted. Moreover, female individuals’ fairness norm enforcement was influenced by group affiliation; that is, unfair offers from ingroups were more likely to be accepted by female young adults than those from outgroups. In some ways, the direction of this group bias was in accordance with social identity theory, which implies that positive evaluations induced by group identity can forgive ingroup perpetrators [23,24]. Most importantly, we found that ingroup identification could exacerbate ingroup favouritism during fairness norm enforcement, especially for moderately unfair offers. In agreement with our findings, Kubota et al. (2013) measured participants’ implicit race bias and investigated whether negative race associations could predict race bias in rejecting unfair proposals from black relative to white proposers [12]. They found that participants rejected more unfair proposals from black than from white players, and this pattern was reinforced by implicit racial bias. In other words, race-based ingroup favouritism during fairness norm enforcement is significantly predicted by implicit race bias. These findings also support our hypothesis.

## 3. Study 2

Using a small sample laboratory design, Study 1 provides causal evidence for our hypothesis that higher ingroup identification is more likely to show stronger ingroup favouritism. To replicate and extend this finding in a moderate sample (*N* = 332), Study 2 recruits college students from the author’s university. We also measure participants’ allocation expectations for ingroup and outgroup proposers in a hypothetical one-shot ultimatum game and further investigated the possible mechanisms (namely, *positive expectations*) behind the connection between ingroup identification and group favouritism during fairness enforcement.

### 3.1. Participants and Procedure

#### 3.1.1. Design and Participants

The study adopted a 2 (ingroup identification: high vs. low) × 2 (group affiliation: ingroup vs. outgroup) mixed factorial design. Questionnaires were conducted by convenience sampling and cluster sampling methods. The participants were college students of different disciplines in two universities in Xinxiang City, Henan Province, who completed the questionnaires during recess. Our self-reported data were collected anonymously and voluntarily to prevent social desirability and response bias. Specifically, participants were informed that their data would be used for scientific research and not be associated with their academic performance and evaluation. If they feel uncomfortable, they could leave at any time. The final number of questionnaires collected was 358. The participants were randomly assigned to either the high or low group identification condition, with 179 participants in each group. Approval for this study was obtained from the ethics committee of the Faculty of Education, Henan Normal University.

#### 3.1.2. Procedure and Measures

In this procedure, four parts were considered: demographic information, manipulation of group affiliation, manipulation checks and a hypothetical one-shot ultimatum game. The demographic information included age, gender and whether the participant was an only child.

First, our approach to manipulating group affiliation and ingroup identification was based on a minimal group paradigm [31]. Specifically, upon completion of the eight-item Justice Sensitivity Inventory [50], the participants were instructed to imagine two unknown students to complete the subsequent interaction, one who answered similarly to them and the other who responded differently from them. The former pertained to the ingroup condition, whereas the latter referred to the outgroup condition. Moreover, we used the degrees of similarity to manipulate participants’ ingroup identification. In particular, we further encouraged participants to imagine completing the interaction with someone *very* similar or *moderately* similar (high vs. low group identification, respectively).

Second, to check our manipulation of group affiliation and ingroup identification, the participants needed to indicate whether the other student responded similarly to their own questionnaire (*very/moderately similar* or *dissimilar*). Furthermore, each participant completed the Inclusion of Other in the Self Scale (IOS) [51], which consisted of five pairs of circles varying in their degree of interlink, ranging from 1 (*no interlink*) to 5 (*extreme interlink*). They should choose the pair that best described their level of social distance with their imaginary ingroup and outgroup counterparts.

Finally, we adopted an intergroup version of the ultimatum game that was used in a previous study [4]. The participants were asked to imagine completing the interaction with a similar person or a dissimilar person. Each interaction included two questions for the participants on the following topics: (1) *allocation expectation* (AE), i.e., estimates of how much you can expect from the proposer; and (2) *acceptance possibility* (AP), i.e., the likelihood that the participants will accept a moderately unfair offer (7:3 offer) on a scale from 0 to 100% [52]. The reason we chose this proposal was that it had the strongest ambiguity [17,53,54], and the largest effect in Study 1. Last, we also calculated the difference in AE scores and AP scores between the ingroup and outgroup interactions. The former reflected an individual’s *ingroup positive evaluation* (IPE), and the latter reflected the similar *ingroup favouritism score* (IFS) in Study 1. This allows us to explore the mediating role of positive expectation in the relationship between ingroup identification and ingroup favouritism.

### 3.2. Results

#### 3.2.1. Participant Characteristics

Sixteen participants from the high identification condition and ten participants from the low identification condition were excluded due to missing data or identical responses. Ultimately, 332 valid participants were obtained, including 270 women and 288 only children, with an age range of 17 to 23 (*M* = 19.44, *SD* = 0.88) years. The numbers of participants in the high versus low identification groups were 163 versus 169, respectively. Table 1 presented a demographic and justice sensitivity description for the two groups. The groups did not differ in terms of the only child or justice sensitivity from the victim, observer and beneficiary perspectives. The high identification group showed a younger average age, a greater proportion of females, and a higher justice sensitivity from the perpetrator perspective.

#### 3.2.2. Manipulation Checks

**Figure 2:**
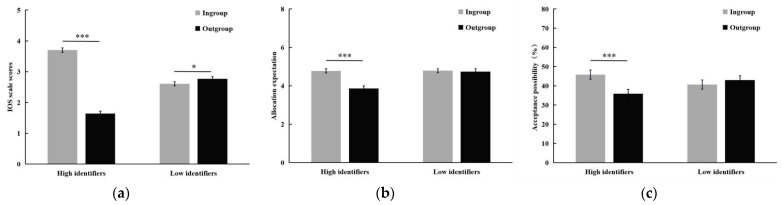
Results from Study 2. Averaged (**a**) IOS scale scores, (**b**) allocation expectation and (**c**) acceptance possibility as a function of group affiliation, separately for the high and low identifiers. Error bars indicate standard error. * *p* < 0.05, *** *p* < 0.001.

In all cases, the participants correctly identified their group membership and identification condition. Moreover, a 2 (ingroup identification: high vs. low) × 2 (group affiliation: ingroup vs. outgroup) ANOVA on the IOS scale scores revealed a significant main effect of group affiliation, *F*(1, 330) = 425.29, *p* < 0.001, partial η^2^ = 0.56, indicating that the participants perceived ingroup members (*M* ± *SE*: 3.15 ± 0.05) as closer socially than outgroup members (*M* ± *SE*: 2.21 ± 0.05). The interaction of ingroup identification × group affiliation was also significant, *F*(1, 330) = 587.54, *p* < 0.001, partial η^2^ = 0.60. A simple test found that high identifiers perceived ingroup members (*M* ± *SE*: 3.70 ± 0.07) as closer socially than outgroup members (*M* ± *SE*: 1.64 ± 0.07), *p* < 0.001, whereas outgroups (*M* ± *SE*: 2.77 ± 0.07) were perceived as more intimate by low identifiers than ingroups (*M* ± *SE*: 2.60 ± 0.07), *p* < 0.05 (see Figure 2a). This two-way interaction was still significant after controlling for demographic variables and justice sensitivity traits, *F*(1, 323) = 553.67, *p* < 0.001, partial η^2^ = 0.63. Hence, it appears that we have successfully manipulated group affiliation.

#### 3.2.3. Allocation Expectation

A 2 (ingroup identification: high vs. low) × 2 (group affiliation: ingroup vs. outgroup) ANOVA on the allocation expectation found a significant main effect of group affiliation, *F*(1, 330) = 24.66, *p* < 0.001, partial η^2^ = 0.07, indicating that participants expected a higher allocation amount from ingroups (*M* ± *SE*: 4.78 ± 0.08) than from outgroups (*M* ± *SE*: 4.30 ± 0.10). The main effect of ingroup identification was significant, *F*(1, 330) = 8.94, *p* < 0.01, partial η^2^ = 0.03, as high identifiers (*M* ± *SE*: 4.32 ± 0.11) expected a lower allocation amount than low identifiers (*M* ± *SE*: 4.76 ± 0.10). The interaction of ingroup identification × group affiliation was also significant, *F*(1, 330) = 20.07, *p* < 0.001, partial η^2^ = 0.06. A simple test found that high identifiers had higher allocation expectation for ingroups (*M* ± *SE*: 4.78 ± 0.11) than for outgroups (*M* ± *SE*: 3.86 ± 0.14), *p* < 0.001, whereas low identifiers showed no significant differences in their distributional expectation between ingroups (*M* ± *SE*: 4.79 ± 0.10) and outgroups (*M* ± *SE*: 4.74 ± 0.14), *p* > 0.05 (see Figure 2b). This two-way interaction was still significant after controlling for demographic variables and justice sensitivity traits, *F*(1, 323) = 17.11, *p* < 0.001, partial η^2^ = 0.05.

#### 3.2.4. Acceptance Possibility

A 2 (ingroup identification: high vs. low) × 2 (group affiliation: ingroup vs. outgroup) ANOVA on the acceptance possibility revealed a significant main effect of group affiliation, *F*(1, 330) = 15.94, *p* < 0.001, partial η^2^ = 0.05, indicating that people are more willing to accept unfair offers from ingroups (*M* ± *SE*: 43.26 ± 1.68%) than from outgroups (*M* ± *SE*: 39.47 ± 1.58%). The interaction of ingroup identification × group affiliation was also significant, *F*(1, 330) = 42.16, *p* < 0.001, partial η^2^ = 0.11. A simple test found that unfair offers from ingroups (*M* ± *SE*: 45.84 ± 2.40%) were more prone to be accepted by high identifiers than those from outgroups (*M* ± *SE*: 35.89 ± 2.25%), *p* < 0.001, whereas group membership did not affect the low identifiers’ acceptance possibility for unfair offers, *p* > 0.05 (see Figure 2c). This two-way interaction was still significant after controlling for demographic variables and justice sensitivity traits, *F*(1, 323) = 39.31, *p* < 0.001, partial η^2^ = 0.11.

#### 3.2.5. The Relation between Ingroup Identification, IPE and IFS

The descriptive statistics of the relevant variables (namely, ingroup identification, ingroup positive expectation and ingroup favouritism score) were presented in Table 2, along with the bivariate correlations. Ingroup identification was positively related to ingroup positive expectation and ingroup favouritism score, and ingroup positive expectation was positively correlated with ingroup favouritism score (all *p*s < 0.001).

To investigate whether the obtained higher ingroup favouritism of moderately unfair proposals among individuals with high ingroup identification could be explained by their increased ingroup positive expectation, we adopted the PROCESS 4.0 macro (Model 4) by Hayes et al. (2017) [55] to conduct a mediation analysis. As predicted, ingroup identification positively predicted ingroup positive expectation and ingroup favouritism score. After controlling for ingroup identification, ingroup positive expectation positively predicted ingroup favouritism score. Moreover, the indirect effect of ingroup identification on ingroup favouritism score through ingroup positive expectation was significant, *b* = 0.05, 95% CI [0.02, 0.09] (see Table 3). Twenty percent of the overall effect was attributable to the mediation effect.

### 3.3. Discussion

The principal finding of Study 2 is that high identifiers show stronger ingroup favouritism than low identifiers and are more willing to accept moderately unfair offers from ingroups. Moreover, high ingroup identification leads to a more positive cooperation expectation for ingroups compared to outgroups, which causes stronger ingroup favouritism for an offer of 7:3. These findings support the interpretation that individuals’ positive evaluation of ingroups could promote forgiveness of ingroup perpetrators. In our view, high expectations of the ingroup are a reflection of positive evaluations, thereby overriding the negative effects induced by ingroup members’ violations. To be more specific, social identity theory argues that people tend to evaluate their group and members positively when they have a clear sense of belonging. This positive assessment of one’s own group might drive people to tolerate and forgive ingroup members’ transgressions. Therefore, despite high expectations of the ingroup’s generosity leading to anticipatory violations and negative emotions, it might still counteract these negative effects, motivating people to forgive the ingroup’s selfishness. Overall, this study replicates the results of the first study in a moderate sample, thereby enhancing the robustness of its findings.

## 4. General Discussion

Across two experiments, we investigate how adults’ ingroup identification enhances ingroup favouritism in fairness enforcement by adopting the incentivized (Study 1) and hypothetical (Study 2) ultimatum game. College students showed ingroup favouritism in both studies, as they were more likely to accept unfair proposals from ingroup members than from outgroup members. Moreover, the participants with high ingroup identification reported stronger ingroup favouritism to the ingroup’s selfishness relative to the outgroup’s selfishness, and the increased positive expectations for ingroup members were partly responsible for this effect. These findings indicate that differences in the degree of ingroup identification have important implications for individuals’ reactions to unfair treatment from their own group.

In accordance with the research hypotheses, group affiliation influences people’s perception of, judgement of and reactions towards others, manifesting in the more preferential and positive treatment of ingroup members. The findings indicate that individuals tend to feel more intimate with ingroups, have stronger positive expectations towards ingroups and punish them less than they punish outgroups for transgressions. A very interesting phenomenon is that people expect higher amounts from ingroup playmates but are willing to accept unfair proposals from them. It is apparent that these results support social identity theory [23] and two meta-analytical studies [33,34], which consider that group integration produces positive evaluations and preferences for one’s own group, resulting in weak punishment for ingroup wrongdoers [5,24]. These findings were also in line with previous empirical research that found that people expect higher allocation amounts [3,18,25], and reject fewer selfish proposals from ingroups or close others [11,12]. These results might be explained from the perspective of group characteristics. On the one hand, individuals usually place more emphasis on group interests and prioritize ingroup cohesion [18,44]. Some studies found that people often adopted norm-conforming behaviours to accept their own group’s unequal proposals, which was critical for maintaining group cohesion [4,15,17]. On the other hand, according to a socioecological perspective [56], the tendencies of Chinese culture emphasize harmonious, cooperative relationships within groups. It may be the result of a long-term strategy to maintain value relationships that the Chinese are more tolerant of ingroups’ transgressions [18,19]. Therefore, maintaining valuable relationships and group cohesion might contribute to ingroup preference during fairness norm enforcement.

In addition, the current study also investigates the enhancing effect of ingroup identification on the group effect on fairness enforcement and potential mechanisms underlying the relationship between ingroup identification and ingroup favouritism. On the one hand, our findings demonstrate that ingroup identification could exacerbate ingroup favouritism both in hypothetical and incentivized ultimatum games, especially for moderately unfair proposals. As predicted by the current study, this finding can be explained by the characteristics of high identifiers and the ambiguity of mild proposals. The group-based ultimatum game required participants to reconcile the differences between fairness principles and group preferences, which directly affected the way people responded to selfishness. Group-oriented individuals are dedicated to maintaining cohesiveness within their groups and focus on group interests. In response to unfair proposals, they give more weight to group preferences, leading to strong ingroup favouritism. Collectivism and group priority play a greater role in Eastern cultures, potentially reinforcing this effect [18,44]. This is consistent with previous research that has found that implicit race bias is a significant and positive predictor of race-based ingroup favouritism during ultimatum games [12]. Moreover, individuals’ responses to moderately unfair proposals vary a great deal because of their ambiguity and weak contextuality [4,57]. In other words, an individual’s group identity is more likely to affect group bias in circumstances that are ambiguous and explainable. On the other hand, we also confirmed that increased ingroup positive expectation can mediate the association between ingroup identification and ingroup favouritism, especially for an offer of 7:3. In other words, people with higher identification tend to have higher ingroup positive expectations, resulting in stronger ingroup favouritism for moderately unfair offers. This result is in line with previous evidence that people who have a high ingroup identity tend to evaluate ingroup members positively and expect more resources from ingroups [3,25] and thus forgive ingroup perpetrators [4,11,12].

There are several limitations to the current study as well. First, because only the minimal group paradigm is used and no real group cues are used to manipulate group relationships, the results are somewhat limited in their external validity. Second, this study looks only at situations in which participants are directly affected by unfair conditions and does not explore unaffected third-party situations. Researchers have also discovered that there is a group bias in third-party punishments [58,59,60]. Therefore, it may be interesting to examine whether the present findings hold up when applied to third parties in the future. Third, the results obtained in study 1 for study 2 are only for female participants, which limits the research. It is therefore necessary for future research to validate the current findings in male populations. Lastly, as in previous studies [3,4,26], the participants’ cooperative expectations were measured before the interaction, and these expectations had an important effect on fairness norm enforcement. Nevertheless, the role of cognitive and emotional factors caused by encountering an injustice, such as perceptions of unfairness and anger, were not taken into account. Recent studies have found that negative emotional responses and unfairness perceptions of unfair distribution are associated with higher levels of rejection and punishment [61,62], so future research should explore how cognitive-emotional factors may impact this behaviour.

## 5. Conclusions

To conclude, the current research finds a salient ingroup favouritism phenomenon and the enhancing effect of ingroup identification on this phenomenon during both incentivized and hypothetical one-shot ultimatum games. When faced with unfair situations, college students show obvious ingroup favouritism; that is, they are more inclined to accept unfair proposals from ingroup than from outgroup members. Moreover, unfair offers from ingroups are more prone to be accepted by high identifiers than those from outgroups, whereas responses of low identifiers to unfair proposals are not affected by group affiliation. In addition, higher identifiers are associated with higher ingroup positive expectations, which then results in greater ingroup favouritism for moderate unfairness. Hence, it is clear that ingroup identification is crucial to shaping people’s reactions when dealing with unfair treatment during intergroup interactions.

## Figures and Tables

**Table 1:** Sample characteristics in Study 2.

	High Identifiers (*N* = 163)	Low Identifiers (*N* = 169)	Statistics
Demographics			
Age	19.31 ± 0.85	19.57 ± 0.88	*t*(330) = −2.68 **
Gender (M/F)	140/23	130/39	*χ*^2^(1) = 4.39 *
One child (O/N)	139/24	149/20	*χ*^2^(1) = 0.60
Justice Sensitivity Inventory			
Victim perspective	2.62 ± 0.84	2.45 ± 0.86	*t*(330) = 1.75
Observer perspective	2.64 ± 0.77	2.50 ± 0.85	*t*(330) = 1.58
Beneficiary perspective	2.24 ± 0.89	2.19 ± 0.82	*t*(330) = 0.50
Perpetrator perspective	3.59 ± 0.99	3.33 ± 1.02	*t*(330) = 2.31 *

Note: Gender and only child were dummy variables coded as 0 = female and 1 = male; 0 = not an only child and 1 = only child. * *p* < 0.05, ** *p* < 0.01.

**Table 2:** Correlations among the key variables in Study 2 (*N* = 332).

Variable	*M*	*SD*	1	2	3
1. Ingroup identification	0.49	0.50	1		
2. Ingroup positive expectation	0.48	1.83	0.24 ***	1	
3. Ingroup favouritism score	3.68	18.33	0.34 ***	0.31 ***	1

Note: Ingroup identification was a dummy variable coded as 0 = low identification and 1 = high identification. *** *p* < 0.001.

**Table 3:** Results of the mediation model.

Variable	Model 1 (IFS)		Model 2 (IPE)		Model 3 (IFS)	
	*β*	*t*	*β*	*t*	*β*	*t*
Age	−0.01	−0.25	−0.03	−0.54	−0.01	−0.12
Gender	<−0.01	0.03	<−0.01	0.01	<−0.01	−0.03
One child	−0.03	0.60	0.01	0.23	−0.03	−0.67
JS-V	−0.06	−1.06	−0.11	−1.74	−0.03	−0.65
JS-O	0.06	0.91	0.18	2.63 **	0.02	0.26
JS-B	0.10	1.83	−0.05	−0.72	0.11	2.06 *
JS-P	<0.01	0.03	0.03	0.43	<−0.01	−0.08
II	0.28	6.27 ***	0.22	4.14 ***	0.24	5.26 ***
IPE					0.21	4.62 ***
*R* ^2^	0.14		0.08		0.19	
*F*	6.34 **		3.51 **		8.36 **	

Note: Gender, only child and II were dummy variables coded as 0 = female and 1 = male, 0 = not an only child and 1 = only child, and 0 = low identification and 1 = high identification. II = ingroup identification, IPE = ingroup positive expectation, IFS = ingroup favouritism score, JS-V, JS-O, JS-B and JS-P = justice sensitivity from victim, observer, beneficiary and perpetrator perspectives, respectively. * *p* < 0.05, ** *p* < 0.01, *** *p* < 0.001.

## Data Availability

The data presented in this study are available on request from the corresponding author.
